# Tumor Exposomics: A New Paradigm for Individualized Continuous Exposure Monitoring

**DOI:** 10.1002/advs.77568

**Published:** 2026-09-06

**Authors:** Kaicheng Shen, Weiyi Wang, Yang Wang, Yiqiang Wu, Xiaohong Liu, Wei Zhang, Juanjuan Ou

**Affiliations:** ^1^ Sichuan Clinical Research Center For Cancer, Sichuan Cancer Center School of Medicine Sichuan Cancer Hospital & Institute University of Electronic Science and Technology of China Chengdu China; ^2^ State Key Laboratory of Woody Oil Resource Utilization Central South University of Forestry and Technology Changsha China; ^3^ Chongqing Key Laboratory of Trusted Perception and Interaction Technology For Intelligent and Connected Vehicles National University of Singapore (Chongqing) Research Institute Chongqing China; ^4^ Chongqing Institute of Green and Intelligent Technology Chinese Academy of Sciences Chongqing China; ^5^ School of Mechanical Engineering Qinghai University Xining China

**Keywords:** integrated continuous monitoring of internal and external exposures, multimodal data integration, personalized exposure assessment, tumor exposomics, wearable devices

## Abstract

Exposomics provides a systems‐level framework to characterize the environmental exposures experienced across the life course and their biological consequences, offering critical insights into tumor initiation and precision prevention. Advances in sensing technologies, intelligent materials, and data science now enable continuous acquisition of external exposures alongside endogenous molecular and phenotypic responses. In this emerging paradigm, exposure is conceptualized not as an isolated variable statistically associated with disease, but as a temporally structured driver embedded within multiscale biological processes. By integrating multimodal monitoring with AI‐enabled causal modeling, exposomics moves cancer risk assessment beyond population averages toward individualized, dynamically updated exposure‐informed risk assessment. This Perspective highlights key technological directions in external‐internal monitoring integration, intelligent sensing ecosystems, and causal data fusion, and outlines a translational framework aimed at supporting precision cancer prevention and early risk management.

## Graphical abstract

Tumor exposomics integrates continuous monitoring of environmental exposures, endogenous biological responses, and behavioral factors within a unified temporal framework. By combining multimodal sensing technologies with AI‐enabled causal modeling, this emerging paradigm reconstructs exposure‐damage trajectories and supports individualized dynamic risk assessment, early warning, and precision prevention of cancer.

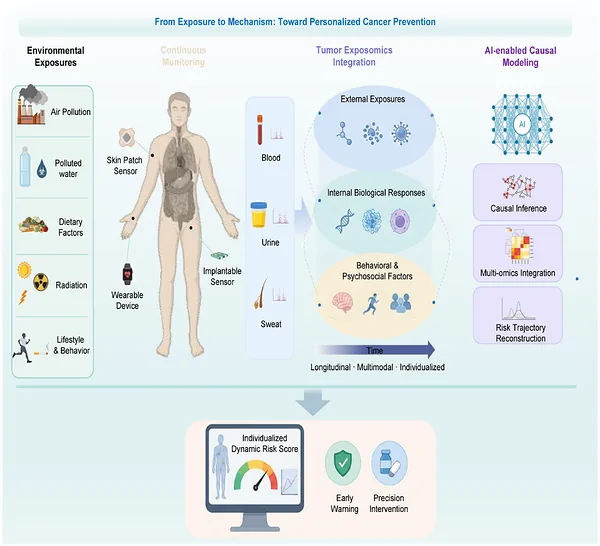

## 1 Introduction

Tumorigenesis represents an extreme outcome of the long‐term interplay between genetic background and environmental exposures. Although genomics has transformed the characterization of inherited cancer susceptibility, nongenetic environmental exposures remain major determinants of cancer development. The concept of the exposome was originally conceived as a comprehensive complement to the human genome, encompassing the cumulative environmental exposures experienced throughout life [1]. This framework has since evolved to emphasize the dynamic interplay between external environmental influences and endogenous biological responses across the life course, extending exposomics from exposure characterization toward a systems‐level understanding of disease etiology [2]. The spatiotemporal heterogeneity of individual genetic contexts, together with interindividual differences in biotransformation and biological response, renders the dissection of exposome‐tumor relationships exceptionally complex and challenging [3, 4]. Compared with early cancer surveillance paradigms that relied predominantly on single‐point measurements, modern exposomics is moving toward dynamic representations capable of capturing long‐latency exposure characteristics [5, 6, 7, 8, 9, 10, 11, 12, 13, 14, 15, 16, 17, 18, 19, 20, 21]. With the continued maturation of high‐throughput analytical platforms, advanced materials, wearable biosensors, and AI‐assisted analytical frameworks, environmental exposures can now be characterized with substantially greater temporal resolution, precision, and biological relevance [22, 23, 24, 25, 26, 27, 28, 29, 30, 31, 32]. Recent advances have enabled continuous and individualized exposure assessment through the integration of wearable sensing technologies and artificial intelligence, and these developments have been comprehensively reviewed elsewhere [33, 34]. Nevertheless, existing frameworks remain largely centered on exposure characterization and predictive modeling, with limited emphasis on continuously integrating multidimensional environmental exposures with endogenous biological responses to elucidate the dynamic mechanisms of tumorigenesis.

The initiation and progression of cancer, as well as many chronic diseases, are profoundly influenced by long‐term environmental exposures, making it difficult to have a clearer understanding of the intrinsic links between exposure sources and disease outcomes. The emergence of exposomics has begun to bridge this critical knowledge gap, parsing the lifelong exposures that underlie tumor development and chronic disease. However, when confronted with complex diseases such as cancer—characterized by long latency periods and multifactorial interactions—traditional “snapshot‐based” research paradigms remain insufficient for capturing low‐dose, long‐term cumulative environmental exposures, which may represent key triggers of early cellular transformation [35, 36].

For example, epidemiological and mechanistic studies have demonstrated that cumulative exposure to specific environmental agents, such as arsenic, cadmium, and particulate matter, is associated with increased risks of multiple cancers through dose‐dependent accumulation of molecular damage, including oxidative stress, DNA damage, and chronic inflammation [37, 38, 39]. However, most current exposure assessments rely on intermittent sampling, occupational records, or population‐level exposure estimates, which provide limited information regarding individual exposure trajectories, temporal fluctuations, and biological responses preceding tumor development. This limitation highlights the need for continuous, individualized exposure monitoring approaches capable of linking dynamic exposure burdens with endogenous damage signatures over time.

Accordingly, there is an urgent need to establish a more comprehensive research paradigm that integrates full‐spectrum exposure histories with real‐time exposure‐induced biological effects, with the aim of constructing disease‐specific risk assessment and therapeutic decision‐support models. Ultimately, such an approach would enable individualized, real‐time exposure characterization and dynamic risk assessment across different stages of cancer development [40, 41]. Notably, the heterogeneity of signatures across different exposure‐induced damage periods may also allow researchers to infer dynamic exposure intensities in reverse, which facilitates deeper mechanistic dissection of stage‐specific carcinogenic processes. This conceptual shift—from conventional association‐based analyses toward causal inference—has the potential to fundamentally reshape the efficiency and precision of cancer prevention and control [42, 43, 44].

Importantly, the application of continuous exposure monitoring may differ substantially depending on the clinical context and intended objective. For cancer prevention and early risk assessment, continuously acquired exposure signals alone cannot capture prior long‐latency exposures and therefore should be integrated with retrospective exposure reconstruction, personal exposure history, and biological indicators reflecting cumulative damage. In contrast, prognostic assessment and treatment monitoring in diagnosed patients would require longitudinal exposure‐response measurements aligned with molecular alterations, therapeutic interventions, and clinical outcomes. Thus, continuous exposure monitoring should not be considered a standalone diagnostic signal, but rather a dynamic data layer that can be integrated with complementary biological and clinical information according to specific cancer‐related applications.

Despite substantial technological progress, important conceptual gaps remain in applying exposomics to cancer research. Current frameworks primarily emphasize exposure characterization and association analyses, with limited integration of temporally synchronized external exposures, endogenous biological responses, and behavioral factors within a unified analytical framework. Moreover, AI‐assisted exposomics has largely focused on prediction, whereas its potential for reconstructing exposure‐induced carcinogenic processes remains underexplored. Against this background, this Perspective proposes a conceptual framework for tumor exposomics that integrates continuous external–internal exposure monitoring with AI‐enabled causal analysis. By establishing temporally aligned linkages between environmental inputs, endogenous biological responses, and behavioral dynamics, we aim to advance exposomics from fragmented exposure assessment toward biologically contextualized, individualized cancer risk evaluation and early intervention.

## 2 New Concepts in Exposure Monitoring

Within this conceptual framework, the interpretation and utility of continuous exposure signals depend on the specific objectives of tumor‐related applications. For cancer prevention and early risk assessment, exposure monitoring should be integrated with retrospective exposure reconstruction and biological indicators of cumulative damage, as newly acquired signals alone cannot capture historical exposures accumulated during the long latency period preceding tumor initiation. In contrast, applications in diagnosed patients, including prognostic stratification and treatment‐response monitoring, require longitudinal exposure‐response measurements aligned with molecular alterations, therapeutic interventions, and clinical outcomes. Therefore, continuous exposure monitoring should be viewed not as a standalone diagnostic signal, but as a dynamic information layer that can be integrated with complementary biological and clinical data according to different stages of cancer development.

### 2.1 Integrated External–Internal Exposure Monitoring

The adverse health effects of exposure are not determined solely by external environmental levels; rather, the true pathogenic processes unfold within the body through a series of progressive biological responses. Notably, the development of chronic diseases and tumors is often the result of long‐term, low‐dose, and sustained exposure to environmental sources [22]. Therefore, accurately establishing the relationship between external exposures and internal biological effects represents a critical unresolved challenge in tumor exposomics.

The core concept of integrated external‐internal exposure monitoring lies in placing continuous sensing of external environmental exposures and the synchronous capture of internal biological perturbations within a unified temporal framework. Through time‐aligned data acquisition, this approach aims to elucidate how the cumulative intensity, frequency, and temporal patterns of external exposures are dynamically translated into molecular responses, providing a mechanistic basis for understanding exposure‐driven tumor development. Such longitudinal exposure‐dose‐response mapping may help overcome the limitations of conventional exposure assessment approaches that rely primarily on single measurements and population‐average estimates. This framework does not emphasize integrating all detection functions into a single device; instead, it highlights the coordinated operation of diverse environmental sensors, biofluid monitoring approaches, and multi‐omics analyses along the temporal dimension, enabling a clear distinction between “the presence of exposure” and “the manifestation of exposure‐induced biological effects” [45, 46]. For long‐term low‐dose or intermittent exposures, short‐term fluctuations in external exposure levels may not immediately manifest as clinical abnormalities. In contrast, the persistent accumulation of internal molecular perturbations, DNA damage signals, or inflammatory states is often more closely aligned with the early stages of tumor risk formation [47, 48]. Incorporating both external exposure inputs and endogenous damage signals into a synchronized monitoring system facilitates reconstruction of the continuous spectrum spanning environmental exposure input to the emergence of tumor‐associated pathological alterations, rather than fragmenting this process into isolated analytical nodes (Figure 1).

**FIGURE 1:**
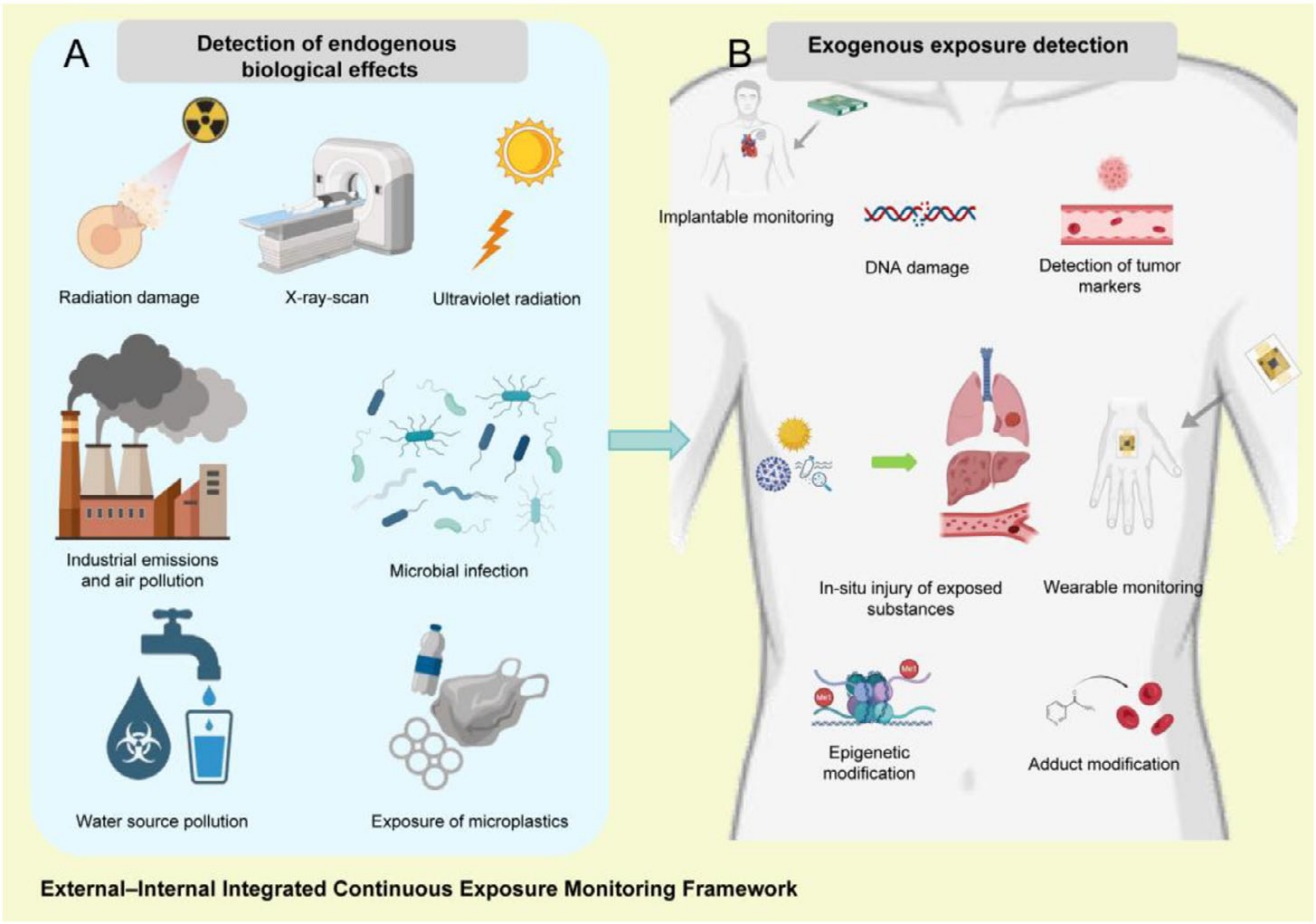
A synchronized exposure monitoring framework is proposed to connect environmental inputs with endogenous biological damage along a unified temporal axis. (A) External chemical and physical exposures, together with individual behavioral rhythms, are continuously captured using portable sensing devices. (B) In parallel, implantable or minimally invasive platforms record internal molecular perturbations, early damage signals, and microenvironmental responses. Through temporal coupling of external exposure signals and internal biological effects, this framework enables continuous, structured reconstruction of exposure–damage relationships across the full exposure trajectory. Some parts of Figure 1 were created with BioRender.com.

### 2.2 Behavior and Psychology: Personalized Monitoring of “Latent Exposures”

Beyond directly measurable chemical, physical, and biological exposures, individuals’ behavioral patterns and psychological states in real‐life environments constitute another form of long‐term, cumulative, and profoundly influential latent exposure. Although such exposures do not manifest as specific material agents, they continuously modulate physiological homeostasis by shaping patterns of environmental contact, activity intensity, and stress background. In recent years, increasing evidence has linked lifestyle factors and mental health conditions—such as circadian disruption, physical inactivity, unhealthy dietary habits, elevated psychological stress, anxiety, and depression—to tumor initiation and progression [49, 50, 51, 52].

In the context of daily diet, in addition to the classic “high‐sugar, high‐fat” Western dietary pattern, ultra‐processed foods (UPFs) have emerged as a more operationalizable dietary exposure phenotype. Repeated studies have demonstrated associations between UPF consumption and increased risks of overall cancer as well as site‐specific malignancies, providing practical evidence that “non‐classical carcinogenic exposures” may promote tumorigenesis indirectly through metabolic dysregulation and chronic inflammation [53, 54]. Consequently, dietary exposure risk monitoring is poised to become a key frontier in future personalized exposure surveillance. This may include real‐time screening of food safety conditions—such as spoilage or expiration—prior to consumption, dynamic early‐warning systems for long‐term high‐dose intake of pathogenic or carcinogenic substances, and the delivery of adjustable, personalized dietary guidance based on continuously accumulated monitoring data—a closed loop from risk identification to behavioral intervention.

From an individual behavioral perspective, environmental contacts are continuously shaped by occupational attributes, lifestyle choices, and behavioral decisions. Prolonged exposure to high‐risk settings—such as environments characterized by dust, radiation, or chemical hazards—or inappropriate intense physical activity under high‐load conditions may induce sustained inflammatory responses and tissue damage states [55, 56, 57, 58]. Such behavior‐driven physiological perturbations may not immediately result in disease but can, over extended time scales, alter tissue microenvironments and create favorable conditions for the accumulation of tumor‐related risk.

Fluctuations in psychological state intervene deeply in this process by modulating stress‐response pathways. Factors such as insomnia, anxiety, and depression can lead to chronic psychological stress or emotional imbalance, triggering persistent stress responses, activating chronic inflammation, and disrupting immune function, ultimately weakening immune surveillance against abnormal cells [59, 60, 61]. Behavioral and psychological factors are tightly coupled: psychological stress can drive maladaptive behavioral patterns, while behavioral imbalance further exacerbates physiological and psychological burdens, forming a self‐reinforcing risk loop [62, 63].

At a collective scale, shared occupational patterns, lifestyle structures, and psychosocial stress backgrounds generate distinct exposure architectures across groups. Continuous monitoring of behavioral trajectories and psychological stress states, when integrated with inflammatory and immune‐related biomarkers, shifts risk assessment to evolve from binary exposure classification toward dynamic modeling of cumulative vulnerability. Personalized characterization of these latent exposures provides a biologically grounded extension of traditional environmental monitoring (Figure 2).

**FIGURE 2:**
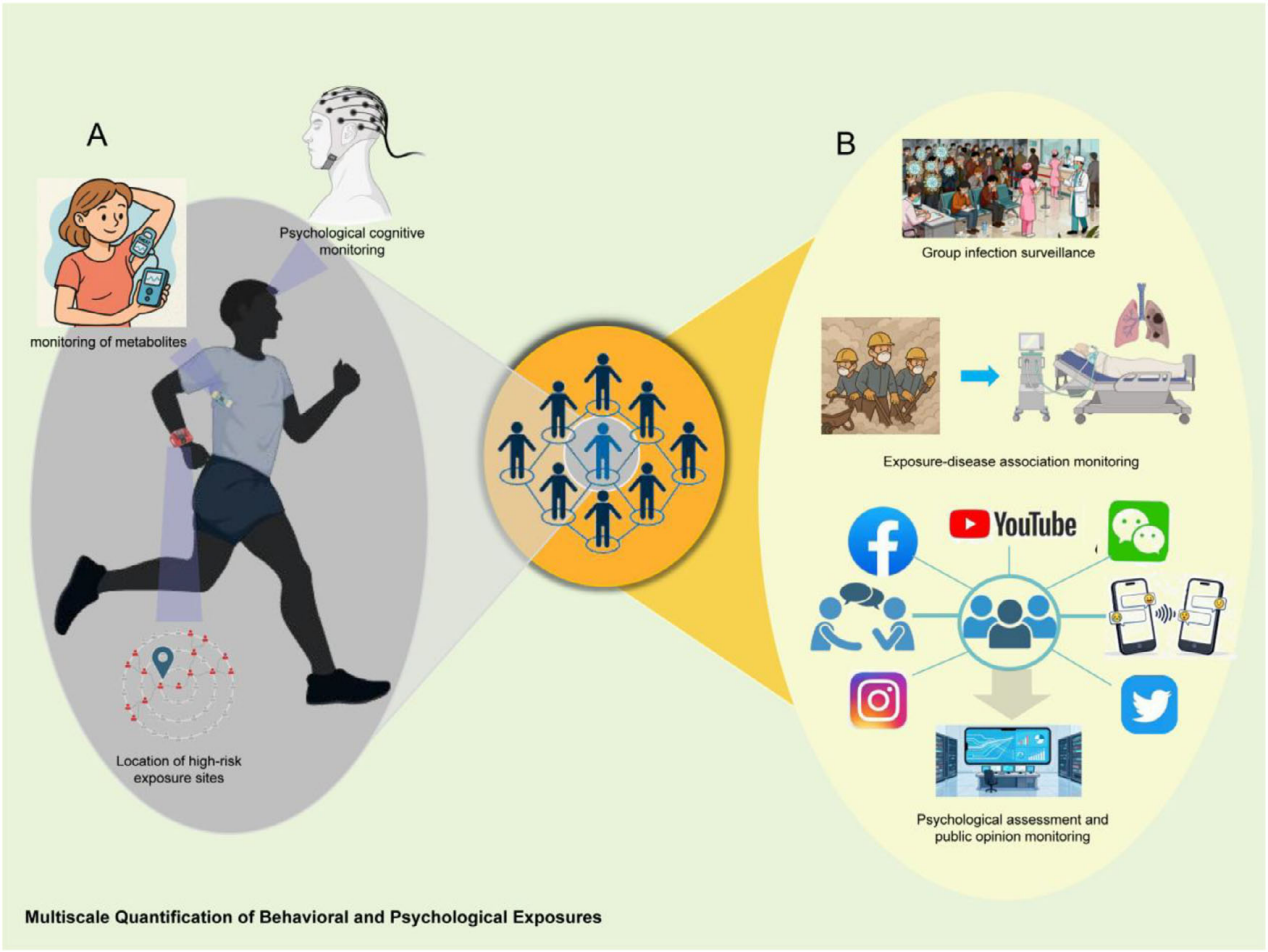
Behavioral patterns and psychological states are conceptualized as latent yet quantifiable exposures that dynamically modulate tumor susceptibility. (A) At the individual level, wearable and intelligent devices continuously monitor activity rhythms, stress‐related signals, and physiological responses, translating daily behaviors into exposure‐relevant metrics. (B) At the population level, aggregated digital epidemiology data capture shared psychosocial exposure landscapes shaped by occupation, mobility, and collective emotional states. Integrating individual and population‐scale signals transforms latent behavioral and psychological exposures into dynamic, biologically grounded risk trajectories. Some parts of Figure 2 were created with BioRender.com.

## 3 Constructing a Continuous Exposure Sensing Network

Traditional exposure assessments have largely been confined within laboratory settings, relying on costly, low‐frequency professional sampling. However, to truly decipher the complex spatiotemporal dynamics underlying tumorigenesis, monitoring must extend into the individual's real‐life environment. Future exposure surveillance will transcend the mere aggregation of discrete devices, evolving into a seamless “continuous exposure ecosystem” embedded within daily life. Within this network, physical spaces, biological interfaces, and digital behaviors will be redefined, collectively forming a multidimensional data infrastructure for elucidating the evolution from exposure to disease [64]. Given the early stage of tumor exposomics, the sensing technologies discussed in this section vary considerably in their level of maturity. While some have already reached practical application, others remain at the proof‐of‐concept stage. They are included because each represents a potentially important step toward continuous, multidimensional exposure monitoring, although their readiness for clinical translation differs substantially. Importantly, the value of continuous sensing lies not only in increasing measurement frequency but also in enabling reconstruction of individualized exposure trajectories. For many environmentally associated cancers, disease risk is unlikely to be determined by a single exposure event but rather by cumulative burden, temporal patterns, and biological susceptibility [65]. Therefore, future monitoring systems should prioritize longitudinal exposure‐dose‐response integration rather than isolated detection of individual exposure events.

However, the real‐world feasibility of exposure monitoring technologies is also shaped by healthcare systems, reimbursement policies, and regional differences in digital health adoption. While the deployment of advanced sensing platforms may remain limited in some settings due to cost, accessibility, and regulatory barriers, other healthcare systems have begun integrating consumer‐grade wearable devices into preventive and remote monitoring programs. For example, reimbursement initiatives and digital health frameworks in some regions have supported the clinical adoption of wearable‐based health monitoring [66]. Therefore, the translational pathway of tumor exposomics will likely depend not only on technological advancement but also on healthcare infrastructure, economic models, and societal acceptance.

### 3.1 Constructing “Outposts” for Intelligent Exposure Monitoring

The individual living environment represents the location of the highest frequency of interaction between the individual and the external world, as well as the most intensive accumulation of exposure. Future smart home systems should not merely serve the convenience of daily life but must be reshaped as the first “immune defense line” against environmental carcinogenic risks [67, 68].

The essence of this transformation lies in rendering monitoring technologies “invisible” and “normalized.” For instance, as water and diet are the primary channels for external chemicals entering the body, their monitoring should not rely on users actively sending samples for testing. Smart water purification systems are poised to evolve into household water quality sentinels. By integrating multimodal spectral and electrochemical sensing modules—such as miniaturized spectrometers based on metasurfaces or quantum dot sensors—combined with multifunctional adsorbent materials like molecularly imprinted polymers (MIPs) and metal–organic frameworks (MOFs), these systems can perform in situ, real‐time “background scanning” for heavy metals, microplastics, and complex organic pollutants [29, 69, 70, 71, 72, 73, 74, 75, 76, 77]. The value of this monitoring lies in its ability to capture transient fluctuations in water contamination, a blind spot often missed by traditional periodic spot checks.

Similarly, the digital reconstruction of dietary exposure will transcend simple caloric counting. Future smart tableware and kitchen ecosystems will use spectral recognition to rapidly screen for nutritional content, harmful additives, and spoilage markers—such as biogenic amines—the moment ingredients are processed or consumed [78, 79, 80, 81]. More importantly, as the exit point for metabolic products, excreta carries definitive information regarding the body's exposure burden. The deployment of smart toilet systems will return the analysis of urine and feces from hospital laboratories to the family bathroom. In a state imperceptible to the user, these systems will establish long‐term baselines for the excretion of exogenous pollutants and fluctuations in endogenous metabolites (such as oxidative stress products) [82, 83, 84]. This passive continuous monitoring model transforms the home from a passive exposure reception field into an intelligent outpost capable of actively identifying risks and recording cumulative doses.

### 3.2 A Panoramic Monitoring Network for the Digital Life Interface

Building upon this foundation, an integrated technology platform based on personalized exposure monitoring serves as the ultimate interface for capturing the endogenous biological effects triggered by exposure. Future monitoring technologies will blur the boundary between biological and digital signals, transforming the human body into a multidimensional biosensing system capable of self‐reporting exposure states. Integrating advanced monitoring technologies is key to achieving comprehensive individualized exposure monitoring. During the contact process between the human body and the external exposure environment, there are various transmission vectors via the mouth, nose, digestive tract, and skin. Future technological advancements must focus on personalized “first contact” monitoring around these aspects, such as microfluidic body fluid capture systems, flexible electronic skins, and hydrogel‐based biointerfaces [85, 86, 87, 88].

In this vision, the respiratory tract assumes the role of the frontier for interaction with the environment; a monitoring system built around the respiratory tract possesses a natural advantage in achieving synchronized perception of “external exposure input—endogenous effect” [89, 90]. Regarding exogenous exposure monitoring, future users will utilize open or semi‐open micro‐sampling carriers, such as smart nose plugs or embedded devices, to continuously capture microbes, aerosols, and particulate matter in the air during daily activities, and deliver early warnings of exposure source contact at the sub‐clinical stage [91]. Conversely, for endogenous responses, integrated smart mask systems will become in situ collection stations for exhaled breath condensate, utilizing attached sensors to continuously analyze metabolic small molecules and inflammatory mediators [92]. This mode of parallel acquisition of “inhalation exposure” and “exhalation response” within the same physiological channel clearly constructs the core path for portable systems to achieve integrated internal–external perception. Although these wearable microfluidic and respiratory sensing platforms have demonstrated promising analytical performance in laboratory and pilot studies, further improvements in long‐term stability, calibration robustness, and clinical validation will be required before widespread deployment.

Beyond flowing gaseous media, fluid media, especially sweat, occupy a core ecological niche in constructing continuous monitoring maps. Due to its non‐invasive and continuously accessible nature, sweat possesses a unique dual attribute of “behavior‐physiology”: it is often actively triggered when the body is under high metabolic states such as exercise load, heat stress, or psychological tension, which is precisely the optimal window for assessing the acute impact of environmental exposure on the body [93, 94]. Advanced flexible microfluidics will be dedicated to solving the problem of mixing new and old sweat, making it a dynamic molecular window reflecting shifts in biochemical homeostasis, synchronously capturing metabolic changes and trace inflammatory factors [95]. Simultaneously, as an important supplement to sweat monitoring, smart fabrics or micro‐patches targeting other body fluids—such as residual urine, semen, and vaginal secretions—will extend the monitoring reach to specific microenvironments like the urogenital system, perfecting the three‐dimensional understanding of local tissue exposure risks [46]. By directly integrating sensing functions into the textile substrate itself, smart textiles provide a critical solution for achieving long‐term, unobtrusive, and continuous data acquisition [96].

With the expansion of monitoring media, portable devices carrying these functions are evolving toward multifunctionality, integration, and invisibility [45]. Photo‐responsive or colorimetric sensing elements worn on the surface of clothing will be responsible for real‐time recording of exogenous physical shocks such as ultraviolet rays or ionizing radiation [97]. Meanwhile, multimodal detection units integrated into intimate layers (e.g., underwear) will focus on continuous observation of areas susceptible to subcutaneous tumors, such as realizing “all‐weather screening” for superficial tumors by identifying microcirculation and metabolic thermal abnormalities in breast tissue [98]. In this layered sensing system, mature terminals like smart watches will become the hub for information integration. They will no longer be limited to recording basic vital signs but will aggregate multi‐source data from respiratory masks, sweat patches, and smart clothing, constructing a panoramic monitoring network covering “external exposure input—internal injury response” through edge computing. These wearable sensing platforms have already achieved broad commercialization and are well suited for large‐scale environmental and physiological monitoring, although their capability for molecularly resolved exposure assessment remains relatively limited.

To deeply analyze the exposure–disease chain, future monitoring networks will require further breakthroughs across the skin barrier, extending into the internal biological environment. Although blood represents the biological medium with the highest information density, continuous tracking remains difficult to achieve using conventional approaches. In this context, implantable or semi‐implantable biosensing devices provide a direct and efficient means of accessing internal biological signals, enabling the capture of deep biological effects [99]. Such implantable micro‐detectors are expected to reside subcutaneously or around specific lesions for long periods, performing in situ real‐time dynamic monitoring of low‐abundance tumor markers, DNA damage signals, and chronic inflammatory factors flowing in the blood [100]. Although technical challenges such as biocompatibility and self‐powering technologies remain to be overcome [101], implantable monitoring represents the deep future of exposure science—it will achieve precise alignment with surface portable devices on the timeline, thereby truly completing the continuous analysis of the “exposure—damage—tumor evolution” process. However, Implantable sensing systems remain largely at the proof‐of‐concept stage, with long‐term biocompatibility, power management, and regulatory approval representing major barriers to clinical translation.

### 3.3 Digital Twins and Invisible Exposures: Proxy Representations of Behavioral Trajectories

Apart from physical and chemical exposures, behavioral patterns and psychological states constitute the latent exposure factors of exposomics—crucial yet long invisible. Individual anxiety levels, sleep rhythms, and degrees of social isolation not only directly regulate immune system homeostasis but also profoundly affect the body's susceptibility threshold to external carcinogenic factors. Future exposure monitoring must incorporate this dimension, and digital phenotyping serves as the key proxy for capturing it [102].

We do not need to equip everyone with cumbersome EEG helmets, because an individual's digital footprint—interaction frequency on social media, acceleration trajectories of mobile devices, subtle changes in voice intonation—faithfully maps their internal psychological and cognitive states. Future intelligent monitoring networks will utilize privacy computing technologies to construct an individual's “behavioral digital twin” in the cloud. In this system, frequent mobile phone operations late at night are no longer just usage records but are decoded by algorithms as signals of circadian rhythm disruption; language fragments on social platforms are no longer meaningless characters but are reconstructed into dynamic curves of stress and anxiety levels. The fusion of this digital phenotype with biosensing data will empower us with a novel analytical capability: we will not only see that a person is exposed to a certain environmental pollutant but also judge via their digital behavior whether they are currently in a “high susceptibility window” of compromised immunity. This will fundamentally change the logic of risk assessment, shifting intervention strategies from singular environmental blocking to systemic health management covering psychological regulation and behavioral intervention. Nevertheless, behavioral and digital phenotyping approaches should be considered complementary contextual information rather than universally applicable exposure measurements. Their real‐world implementation will require careful consideration of user acceptance, privacy protection, data governance, and algorithmic validity. Accordingly, these approaches may initially serve targeted populations or research settings rather than being universally adopted as continuous monitoring tools.

In summary, these multi‐form, cross‐scenario monitoring technologies do not exist in isolation but collectively constitute an individualized exposure sensing network that unfolds across time and space dimensions. By simultaneously covering external environmental contact, internal injury response, and novel exposure forms related to behavioral and psychological states, the exposome can shift from measurements derived from single sources or single time points toward a stereoscopic, dynamic representation of the exposure process itself [103]. This integration of information across multiple channels and time scales provides a systemic perspective for understanding how exogenous exposures progressively translate into endogenous biological perturbations, laying the foundation for efficient, low‐burden individualized continuous monitoring. Consequently, exposure monitoring may gradually transition from a research demonstration toward real‐world implementation; however, practical deployment will likely rely on context‐specific combinations of sensing modalities rather than requiring individuals to continuously adopt all available technologies simultaneously (Figure 3) [104]. It should be noted, however, that the technologies discussed in this section represent different stages of development. Continued advances in sensor durability, biocompatibility, multimodal data integration, and regulatory translation will be essential for broader adoption in precision oncology.

**FIGURE 3:**
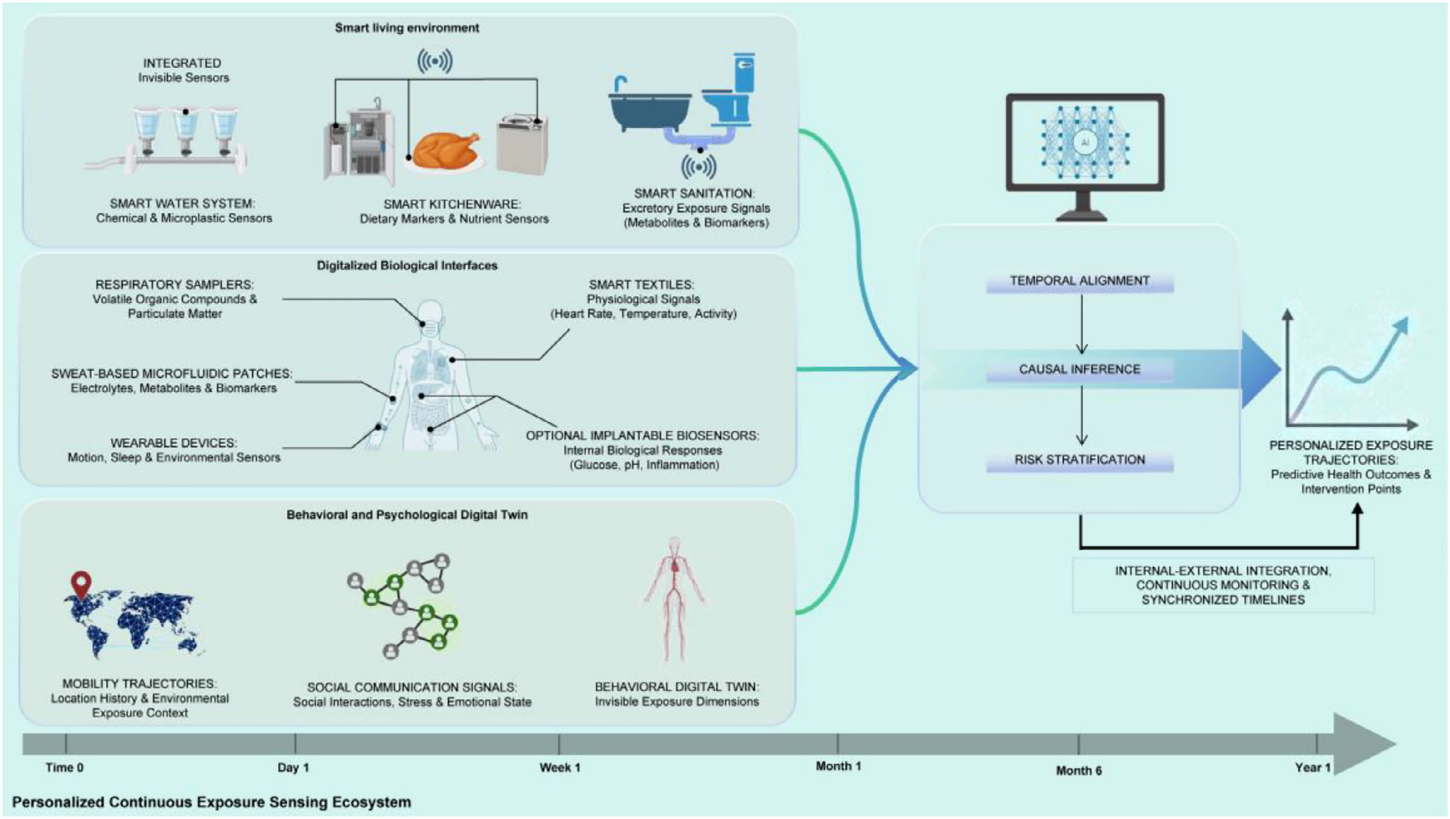
A personalized continuous exposure sensing ecosystem is embedded across daily life scenarios to capture exposure trajectories in real time. Environmental outposts within living spaces monitor external exposure inputs, while wearable and minimally invasive bio‐interfaces synchronously record endogenous molecular responses. In parallel, digital twin models reconstruct latent behavioral and psychological exposures. Through temporal synchronization and AI‐driven integration, fragmented signals are consolidated into continuous, individualized exposure trajectories. Some parts of Figure 3 were created with BioRender.com.

## 4 Intelligent Causal Models and Dynamic Risk Landscape Reconstruction

As multimodal sensing networks spread across life scenarios, the challenge facing exposomics is no longer a lack of data, but rather the “multidimensional heterogeneity” and “spatiotemporal mismatch” of the data [105, 106, 107]. To extract clinical decision‐making evidence from massive and fragmented monitoring data, an AI‐driven central engine must be constructed. This engine is designed to move beyond traditional epidemiology's reliance on statistical correlation, utilizing deep learning and causal inference technologies to reconstruct a definitive causal landscape between “exposure—biological response—disease outcome”.

First, the spatiotemporal alignment of multimodal data is the cornerstone for deciphering exposure chains. Time‐series data from environmental sensors, metabolic rhythms of biochemical indicators in body fluids, and the pathological progression of tumorigenesis naturally possess vast differences in time scales [108]. How to efficiently integrate and analyze these multidimensional data will be a major challenge for the development of AI technology now and in the future.

Therefore, breakthroughs in AI intelligent algorithm technology will no longer focus merely on data stitching, but will instead function as a spatiotemporal integration and alignment framework. In this paradigm, discrete external exposure events—such as high‐concentration inhalation episodes—can be precisely registered with subsequent internal biological perturbations along a shared causal timeline. These perturbations may include the formation of specific DNA adducts, transient peaks in oxidative stress, and other molecular or cellular responses. A central challenge lies in accurately aligning heterogeneous exposure sources in the environment, including dust, smoke, and pathogenic particles, with long‐term and stage‐specific biological perturbations within the human body. Such perturbations span multiple temporal scales and tissue‐specific spatial dimensions, encompassing DNA damage, adduct accumulation, and disruptions in inflammatory homeostasis.

On the basis of this spatiotemporal alignment, how can AI algorithms map environmental exposures and biological responses to the same causal timeline, while integrating multidimensional information such as individuals' daily activities and psychological states, to ultimately achieve individualized and precise monitoring and assessment of exposure damage? Future technological breakthroughs are expected to develop more systematic data fusion paradigms on this basis, allowing originally fragmented multi‐source data to move from “juxtaposed correlation” toward “causal reconstruction.” In this way, scattered “data silos” will be reorganized into temporally structured exposure trajectories with temporal direction and biological significance—offering researchers a new analytical framework to trace how environmental exposures accumulate over time and dynamically translate into biological damage, and laying the theoretical and technical groundwork for individualized exposure early warning and intervention (Figure 4).

**FIGURE 4:**
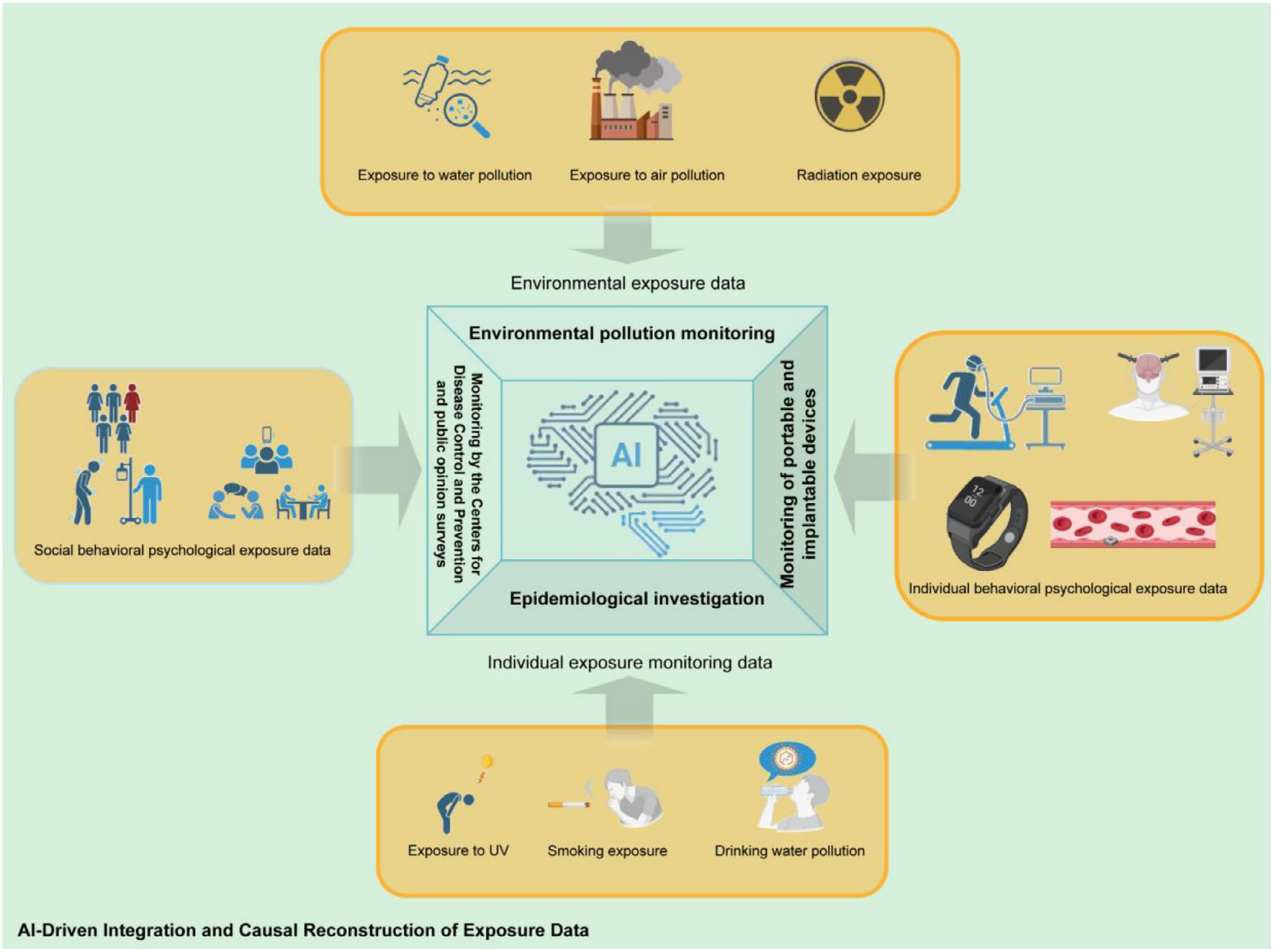
An AI‐centered analytical framework integrates heterogeneous exposure data into a unified causal structure. External environmental measurements, internal biological signals, and behavioral–psychological information are temporally aligned and jointly analyzed across scales. By transforming fragmented multimodal inputs into coherent exposure narratives, this platform enables causal reconstruction of exposure–damage pathways beyond conventional correlation‐based assessment. Some parts of Figure 4 were created with BioRender.com.

On this basis, AI's function will leap from pattern recognition to causal inference and critical window identification. While traditional black‐box models can predict risk, they often struggle to explain mechanisms. A new generation of explainable AI, combined with prior biological knowledge (such as metabolic pathway maps), will be dedicated to disentangling confounding factors in complex exposure networks to identify the “critical exposure windows” that truly drive disease occurrence. This is analogous to precisely locating the “key frame” where a “carcinogenic mutation” occurs in a long life movie—is it because a certain chemical accumulation in childhood weakened the repair capacity, or did long‐term psychological stress in adulthood become the last straw that broke the frontline environmental monitoring node? By constructing counterfactual reasoning models, AI can simulate “how the body state would evolve if this exposure had not occurred,” and validate the causal necessity of exposure and disease mechanistically—not merely correlate them.

Importantly, AI does not eliminate the fundamental challenges of causal inference in exposomics. Continuous multimodal monitoring can substantially improve the temporal resolution and biological context of exposure assessment, yet observational data remain susceptible to residual confounding and other sources of bias. Consequently, AI is best regarded as an evidence‐integration framework that strengthens mechanistic inference and hypothesis generation, rather than a substitute for experimental validation or prospective clinical investigation.

Collectively, this complex computational process will converge into an intuitive clinical tool: an Individualized Dynamic Risk Scoring (iDRS) system. This indicator is no longer based on population average risk, but integrates an individual's long‐term exposure history with real‐time physiological response signals to form a continuously updated risk representation. The model will dynamically fuse multi‐source data streams from home environments, wearable, and implantable devices. When cumulative exposure loads and endogenous damage signals amplify synergistically in time and approach biological thresholds, the system can automatically trigger risk warnings and adjust intervention levels [109, 110, 111]. Consequently, tumor prevention and control is expected to move forward from passive diagnosis after symptoms appear to active intervention during the risk accumulation stage, allowing clinicians to reverse‐infer potential pathological processes based on dynamic risk scores and formulate targeted exposure blocking, metabolic clearance, or immune regulation strategies, delivering the closed‐loop transformation of exposomics from mechanism analysis to clinical application (Figure 5).

**FIGURE 5:**
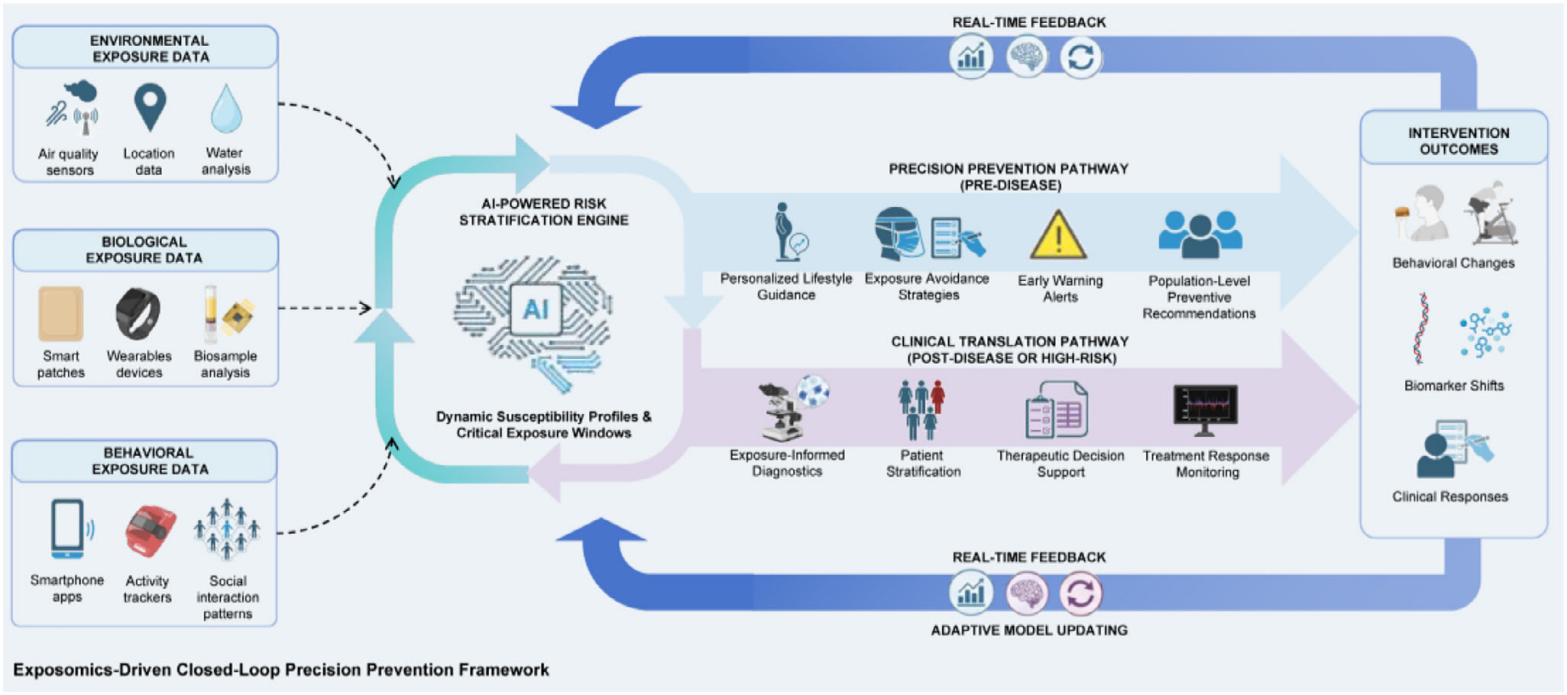
A closed‐loop exposomics framework links continuous exposure monitoring with dynamic risk scoring and clinical intervention. For subclinical individuals, exposure‐informed early warnings guide behavioral and environmental adjustments. For high‐risk or affected populations, exposure fingerprints support clinical stratification and treatment optimization. Real‐time biological feedback following intervention iteratively refines risk models, enabling life‐cycle precision management. Some parts of Figure 5 were created with BioRender.com.

## 5 Exposomics and Tumor Prevention: AI‐Driven Precision Medicine and the Future of Global Collaboration

The ultimate mission of exposomics is not only to explain the world but to change health outcomes through intervention. When multimodal monitoring networks capture data and AI engines analyze risks, a “closed‐loop decision path” from digital insights to medical action must be constructed. Future exposure science will transcend a purely observational role, evolving into a dynamic operating system capable of guiding behavioral interventions in real‐time, reshaping clinical diagnosis and treatment strategies, and driving new drug development.

The core of this system lies in using intelligent sensing networks to obtain individual exposure information in real‐time and performing a comprehensive reconstruction of disease progression paths through dynamic risk scoring models. This model not only improves the efficiency of precision cancer prevention and control but also promotes the exploration of causal relationships between exposure sources and diseases, optimizing individualized treatment plans and shifting medicine from passive treatment to active prevention [112]. For example, targeting the long‐term accumulation of specific environmental pollutants (such as microplastics and persistent organic pollutants) in target tissues like the ovaries or uterus [113], in the future, we will not only be able to detect their dynamic deposition amounts but also decipher the specific pathological perturbation characteristics and tumor microenvironment remodeling patterns induced by the entire exposure process. Meanwhile, during the clinical translation process, as early warning capabilities enhance, diagnostic and treatment strategies can be flexibly adjusted based on exposure history and real‐time changes, achieving a seamless connection from early detection to precision intervention (Figure 5).

In translational contexts, exposomics‐derived risk profiles can guide stratified surveillance strategies and exposure‐targeted intervention. For subclinical populations, adaptive risk modeling supports behavioral modification, environmental mitigation, and metabolic optimization prior to pathological manifestation. For high‐risk or diagnosed individuals, exposure signatures may inform clinical subtyping, therapeutic sensitivity assessment, and longitudinal monitoring of treatment response. Continuous feedback of post‐intervention biological outcomes enables iterative refinement of risk algorithms, forming a responsive decision‐support ecosystem for precision oncology.

Nevertheless, technological progress alone is insufficient to sustain the advancement of exposomics; it faces a series of technical and social challenges. From a technical perspective, current developments in monitoring equipment and sensor technology still face many bottlenecks, such as real‐time synchronization and integration of data, stability and biocompatibility of devices, as well as the capacity for miniaturization and stable long‐term use [114, 115, 116]. Although technology continues to advance, how to break through the timeliness and precision of devices in the data collection, processing, and feedback stages remains a key issue. At the same time, with the development of technology, privacy protection and data security issues have also become important challenges that need urgent solutions. While technology can improve the efficiency of data acquisition and analysis, how to effectively apply it while ensuring data privacy and protecting individual rights is an ethical issue that must be considered for the further popularization of technology.

In the era of information globalization, the progress of exposomics also faces tests of transnational collaboration and social ethics. Transboundary pollution issues such as environmental pollution, radiation, and microplastics require countries to share exposure data and monitoring information, which places extremely high demands on global health governance (Figure 6) [117, 118, 119]. However, due to differences among countries in environmental monitoring, data sharing, and management systems, how to establish effective information exchange and coordination mechanisms remains a major obstacle to the development of global exposomics. Meanwhile, the global gap between rich and poor and differences in the technological foundations of various regions also cause low‐income countries to face greater challenges in the application of exposomics technology. How to achieve global popularization and equitable application of technology is also a problem that needs to be solved in global cooperation [120].

**FIGURE 6:**
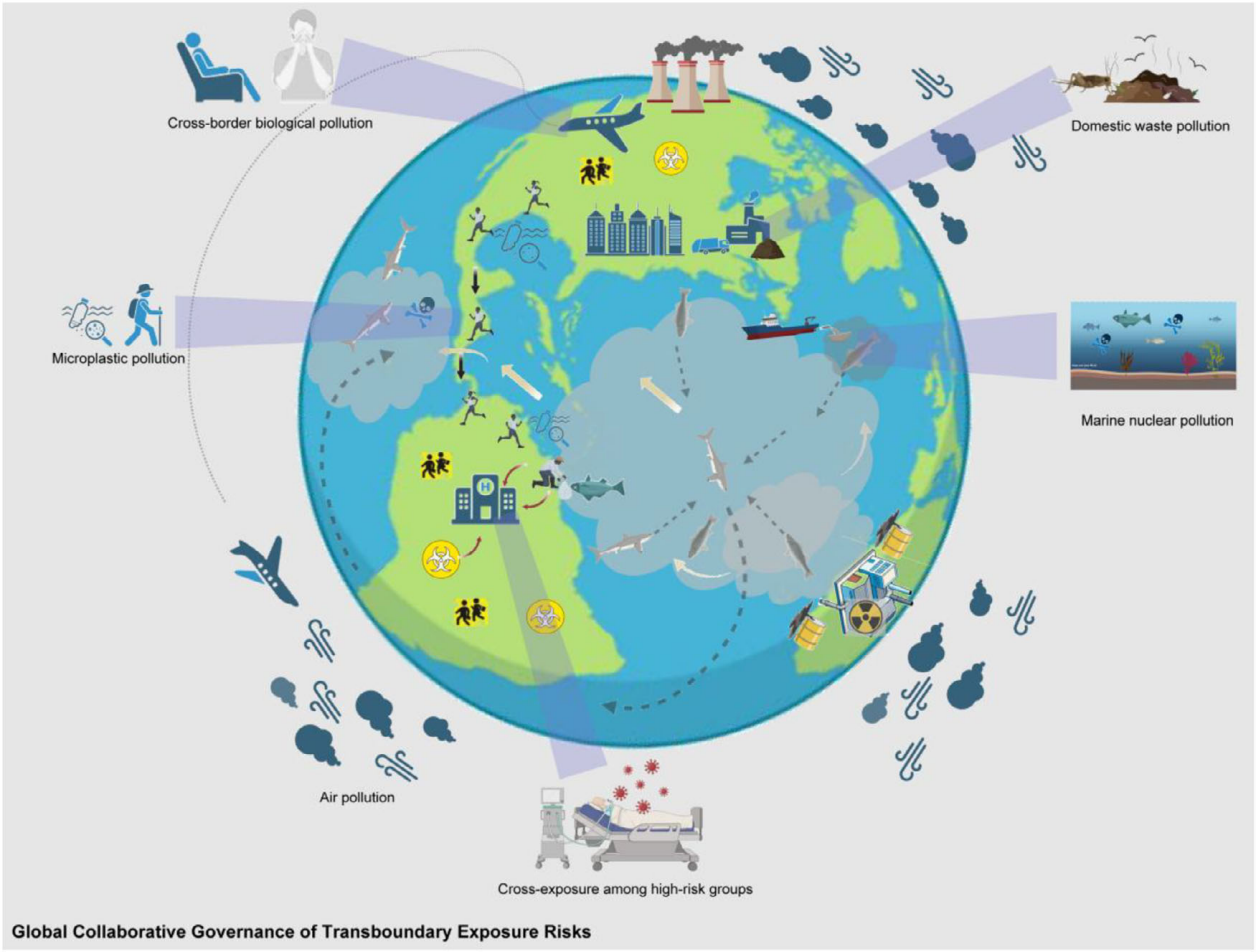
A global collaborative governance framework is proposed to address transboundary exposure risks. By integrating environmental monitoring networks, digital epidemiology platforms, and standardized analytical algorithms, emerging global threats can be tracked in real time. This shared governance model supports coordinated public health decision‐making and promotes equitable application of exposomics technologies across regions. Some parts of Figure 6 were created with BioRender.com.

The implementation of continuous tumor exposomics also requires careful consideration of ethical, privacy, and governance challenges. Because this framework integrates environmental, physiological, and behavioral data collected over extended periods, informed consent should be dynamic, allowing individuals to retain meaningful control over data collection, storage, and secondary use. Privacy‐preserving strategies, such as federated learning and differential privacy, together with clear governance mechanisms for data ownership and access, will be essential to support responsible data sharing. In addition, AI models should be developed and validated using diverse populations to minimize algorithmic bias and ensure equitable risk assessment across different demographic groups. Ultimately, the successful translation of tumor exposomics will depend not only on continuous exposure monitoring and AI‐enabled analytics, but also on governance frameworks that safeguard individual autonomy, ensure equitable data use, and maintain public trust.

Therefore, the future of exposomics relies not only on technological innovation but also on the cooperation of all sectors of society and the support of global governance systems. It is necessary to promote technological breakthroughs, information sharing, the formulation of ethical standards, and the strengthening of global governance through international cooperation, and ensure that technological progress serves not only developed countries but also benefits vulnerable groups worldwide. Through such cooperation, we can establish a safer, fairer, and more effective global exposure monitoring system on the basis of ensuring individual privacy [121, 122].

## 6 Future Perspective

Exposomics is moving beyond static exposure cataloging toward temporally organized and biologically contextualized monitoring ecosystems. The convergence of flexible materials, embedded sensing platforms, and causal AI modeling captures environmental inputs and endogenous biological responses along a shared temporal continuum. In parallel, increasing attention has been directed toward green sensing materials derived from renewable resources—such as lignocellulosic fibers and plant‐based oils—which offer a viable pathway for enhancing the sustainability and environmental compatibility of continuous monitoring technologies [123, 124]. Under this emerging monitoring architecture, households and individuals become distributed sensing nodes, while intelligent systems translate exposure fingerprints into interpretable and predictive health insights. Importantly, continuous exposure monitoring should be viewed as a complementary longitudinal information layer rather than a replacement for established diagnostic, prognostic, or therapeutic evaluation strategies. Its primary value lies in linking dynamic environmental exposures with endogenous biological responses across different stages of cancer development, thereby providing additional context for individualized risk assessment and clinical decision‐making.

The realization of this vision relies not only on technological maturation but also on robust ethical governance, data security frameworks, and inclusive global collaboration. As environmental risks increasingly transcend national boundaries, exposomics must develop into a cooperative scientific infrastructure capable of integrating molecular‐level evidence with public health decision‐making. By aligning technological innovation with social responsibility, exposure science can play a substantive role in advancing sustainable cancer prevention and strengthening global health resilience.

## Author Contributions

**Xiaohong Liu**: writing – original draft, writing – review and editing, project administration, funding acquisition, resources. **Weiyi Wang**: writing – original draft. **Yang Wang**: writing – original draft. **Wei Zhang**: writing – review and editing, writing – original draft, project administration, resources. **Yiqiang Wu**: writing – original draft, funding acquisition. **Juanjuan Ou**: writing – original draft, writing – review and editing, project administration, resources, funding acquisition. **Kaicheng Shen**: writing – original draft.

## Conflicts of Interest

The authors declare no conflicts of interest.

## Data Availability

The data that support the findings of this study are available from the corresponding author upon reasonable request.
